# Light People: Prof. Donal D C Bradley (FRS)

**DOI:** 10.1038/s41377-024-01527-w

**Published:** 2024-08-06

**Authors:** Ruidong Xia, Ying Hu

**Affiliations:** 1 Key Laboratory for Organic Electronics & Information Displays (KLOEID), Jiangsu-Singapore Joint Research Center for Organic/Bio Electronics & Information Displays, Institute of Advanced Materials (IAM), Nanjing University of Posts and Telecommunications, 9 Wenyuan Road, Nanjing, 210023 China; 2 Executive Management College of CHN ENERGY, North District of Future Science City, Changping District, No.7 Binhe Avenue, Beijing, 102211 China

**Keywords:** Optics and photonics, Electronics, photonics and device physics, Organic LEDs, Solid-state lasers

## Abstract

The invention of organic light emitting diodes (LEDs) led to enormous excitement in both academe and industry in the late 1980’s. Flexibility, large area solution processability, roll-to-roll printing, low cost, and environmentally friendly are some of the advantages of organic semiconductor materials, which brought a new horizon for optoelectronics. Together with the achievement of organic solar cells, transistors, lasers, and amplifiers, this has demonstrated potential applications of organic semiconductors in displays, lighting, solar energy generation, electronics, sensing and imaging, and many aspects of photonics. In an enlightened conversation with Light: Science & Applications, Prof. Donal Bradley (FRS), a pioneer in the field, shared his deep insights on past, current, and future exciting developments of organic optoelectronic materials and devices. In particular, he expressed his opinion on the hot topics related to organic optoelectronics research and application, such as the relationship between organic and inorganic semiconductors and the challenge of electrically pumped organic lasers. As a successful scientist, Donal has also been co-founder of several organic optoelectronics innovation companies and research centers and a long-term academic administrator serving as a Head of Department, Centre Director, and Vice-Rector for Research at Imperial College, Head of the Mathematical, Physical, and Life Sciences Division at the University of Oxford, Vice-President for Research at King Abdullah University of Science and Technology and now Vice-President for Research and Innovation at NEOM U and Executive Director of the NEOM Education, Research and Innovation Foundation. Through this interview, we also explore the major roles and events in Donal’s career experience from the invention of the first conjugated polymer LED in the world to the set-up of entrepreneurial companies, from Cambridge to Sheffield, Imperial College, and Oxford, from the UK to overseas, and from the establishment of the Centre for Plastic Electronics in Imperial College to the set-up of the Oxford Suzhou Centre for Advanced Research (OSCAR). Before the end of the conversation, he also shares his interesting story of identifying a new species of Sea Bream, *Acanthopagrus oconnorae* (Bev Bradley’s Bream), named after his mother and wife, while fishing in the Red Sea.

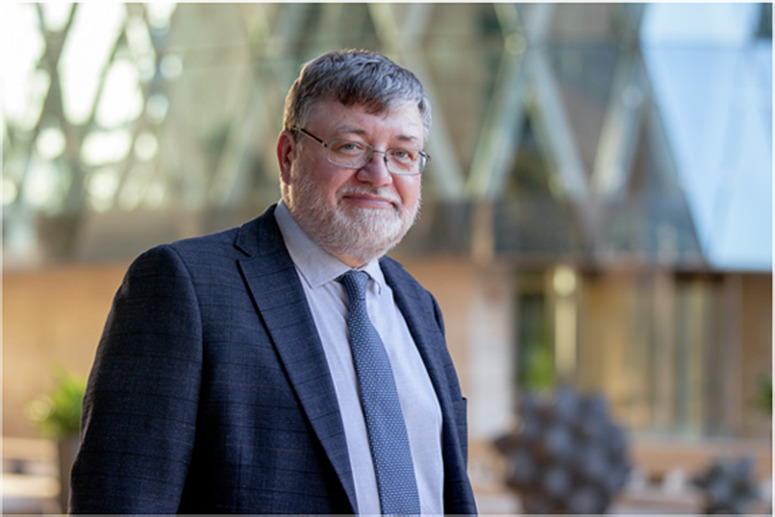



**Biography:** Donal Bradley studied physics as an undergraduate, receiving BSc and ARCS degrees from Imperial College in 1983, and completed his PhD in 1987 at the University of Cambridge on controlling the microstructure and photophysical properties of poly(p-phenylenevinylene) thin films. He also holds honorary DSc degrees from the University of Sheffield (2014) and Hong Kong Baptist University (2017). After completing his PhD, Donal held a Toshiba Research Fellowship at the Toshiba R&D Center in Kawasaki, Japan (1987-88), and a Leverhulme Research Fellowship in Chemical Physics at Corpus Christi College, Cambridge (1987-89). His first faculty position was at the Cavendish Laboratory in Cambridge (Assistant Lecturer, 1989-93), and he subsequently worked at the University of Sheffield (1993-2000; Reader then Professor of Physics), Imperial College London (2000-15; Professor of Solid State Physics, Lee-Lucas Professor of Experimental Physics, Head of the Department of Physics, Director of the Center for Plastic Electronics, Vice-Rector for Research), the University of Oxford (2015-19, Professor of Engineering Science and Physics; Professorial Fellow at Jesus College; Head of the Division of Mathematical, Physical, and Life Sciences), and the King Abdullah University of Science and Technology (2019-22, Distinguished Professor of Materials Physics and Device Engineering; Vice-President for Research), before moving to his current roles at NEOM, where he is Executive Director of the Education, Research, and Innovation Foundation and Vice-President for Research and Innovation at NEOM University. Donal’s research has focused on molecular electronic materials and devices and soluble semiconductors, with an interest in materials and devices for electronics, optoelectronics, and photonics. He is a pioneer in the field, contributing to many scientific advances and innovations, and his work has been recognized by multiple awards and prizes, including the E-MRS Jan Czochralski Gold Medal (2019), Jiangsu Province Governor’s Award for International Cooperation (2016), Founders’ Prize of the IOP Polymer Physics Group (2013), IET Faraday Medal (2010), Royal Society Bakerian Lecture (2010), IOP Faraday Gold Medal (2009), Royal Society Brian Mercer Award (2006), SID Jan Rajchman Prize (2005), ESF European Latsis Prize (2005), EU Descartes Prize (2003), Daiwa Award for Anglo-Japanese Collaboration (1994) and RSA Silver Medal (1983). He is a fellow of the Royal Society (FRS, 2004), US National Academy of Inventors (FNAI, 2020), Institute of Physics (FInstP, 2005), Institution of Engineering and Technology (FIET, 2013), and Royal Society of Arts, Manufactures, and Commerce (FRSA, 1987), an honorary fellow of Churchill College Cambridge (2018), and a chartered engineer (CEng, 2015). Donal was appointed Commander of the Order of the British Empire (CBE) in 2010 by HM Queen Elizabeth II for services to science.

**Q1.** **First, as a renowned researcher in organic optoelectronics, could you please briefly introduce what are organic optoelectronic materials and why they are of interest, especially in comparison with traditional inorganic materials? What are the current and future applications of organic optoelectronic materials and devices?**

Organic materials comprise molecules that are largely composed of carbon and hydrogen, with oxygen, nitrogen, sulfur, and a few other atoms potentially included. These molecular structures comprise a unit, be it a small molecule, a macrocycle, a dendrimer, or a polymer chain within which there is strong covalent bonding holding the atoms together. These molecules can pack to form a solid film with van der Waals, hydrogen, or other types of weak bonding. In addition, the molecular units can be relatively easily dissociated and reconstituted into a form of specific interest, which can be achieved over large areas, if needed, by evaporation, dissolution, or melting. For polymers, this has led to simple processing methods that have driven their ubiquity as structural materials for making objects as diverse as household goods, clothing, and packaging.

For organic molecules to be of interest as electronic, optoelectronic, or photonic materials, we need them to possess the ability to generate interesting electromagnetic responses, basically conductivity; light absorption, emission, and/or amplification; photogeneration of charges or fields; nonlinear displacements in frequency, intensity, or direction; and so on. They also need to provide an advantage over other material systems that demonstrate similar responses, including inorganic elemental/compound, metal chalcogenide, and metal oxide semiconductors. In addition, as advantages are identified, incumbent technologies invariably adapt and evolve to maintain their incumbency, making paradigm changes in materials and devices relatively rare. Typical optoelectronic devices of interest include displays, lighting sources, solar cells, photodiodes, phototransistors, lasers, amplifiers, and electro-optic switches.

The low-temperature and large-area processability of organic materials is one distinction from traditional crystalline inorganic materials, but the desirability of such approaches has driven many subsequent developments to enable the solution processing of inorganic materials, including quantum dots and rods, and to derive low-temperature pyrolysis reactions to generate inorganic films via reactive deposition or the thermal or optical postprocessing of a precursor phase. This is especially the case for metal oxides. Flexibility is another desirable attribute, allowing new form factors, and conformal coating without the need for lattice matching is yet another, which supports heterointegration. On the other hand, the attributes that lead to these properties can impact mechanical and thermal stability.

Organic optoelectronic materials are not new. Two highly successful commercial applications that predate my own involvement in the field are liquid crystal displays (LCDs) and xerographic copiers. The advance of LCDs transitioned the world into the realm of flat-panel, large-area, and portable display devices that became affordable enough for global adoption. They sounded the death knell of the cathode ray tube and led to the first transition of display technology to the Far East, finding their home in Japan at Sharp, Toshiba, Panasonic, Sony, and other companies. Xerographic blends comprising arylamine and other hole transport molecules dispersed in a polymer binder are a charge generation system that allows spatial patterning of a static charge distribution across the surface of a roller. This charge distribution can be used to capture pigment particles that are then thermally fused to paper or other surfaces passing over the roller. The advantages of organic-based systems over previously used inorganic semiconductor photocharge generation layers, such as those containing selenium, include cost and environmental considerations.

Neither LCDs nor organic xerographic systems use these molecular materials as semiconductors. LCDs are based on a refractive index switching phenomenon associated with the field orientation of molecular dipoles that allows ‘gating’ of a light source passed through crossed polarizers, with pixelated filters (initially absorptive) to generate the red, green, and blue elements of the colour gamut. Interestingly, silicon had to, and did, respond to this new need, with first amorphous silicon and then polycrystalline silicon processing being required to produce large-area backplanes to drive the individual liquid crystal shutters that define the desired image. In the xerographic application, the blends are insulators that can hold the photogenerated charges for long enough to copy the information.

For much of the 1960s, 1970s, and early 1980s, it was considered that semiconductors needed to be of ultrahigh purity and crystalline to be useful for device fabrication. Considerable work was done by pioneers of organic optoelectronics, such as Martin Pope, Martin Schadt, Heinz Bässler, and Norbert Karl, on organic crystalline semiconductors such as acenes, arylenes, rylenes, and related molecules, and many device types have been demonstrated, including light emitting diodes, solar cells, optically pumped lasers, photodiodes, and transistors. Thick (by normal semiconductor standards) single organic crystals require very large electric fields to drive devices made therefrom. In addition, a lack of suitable electrode materials led to exotic, often liquid, contacts. It also proved challenging, synthetically, to produce the ultrapure materials that were desired.

The work of Ching Tang and his colleagues at Kodak in the 1980s brought small molecules back into clear view for solar cell and display applications, using organometallic compounds, specifically phthalocyanines and quinolates in bilayer structures. These materials had much more promising performance. They were also not single-crystal structures, a step that helped shift the field towards a more practical fabrication approach. Shogo Saito and Tetsuo Tsutsui progressed these diode structures to three-layer stacks and beyond, and they trained many display industry engineers to build better-performing devices for use in commercial products, initially within Pioneer stereo systems in the automotive sector. OLED displays became a new horizon for organic optoelectronics, yielding another shift in the display manufacturing locus to Korea (LG and Samsung), a further advance in backplane technology (metal oxide transistors), and a strong response from incumbent companies to keep LCDs in the market against a new technology that offered better viewing angle, speed, contrast, colour gamut, and efficiency. Today, OLEDs dominate the display market, with modifications being made to try to upgrade LCDs, for example, adding efficient nitride LED backlights, shifting to fluorescent colour filters, and enhancing the viewing angle with birefringent films.

Conjugated polymers first came into view in the 1970s as synthetic metals and for nonlinear optical refractive index switching in telecoms. A significant step forward in the early 1980s was the development of precursor-route approaches that facilitated solution processing of a nonconjugated intermediate prior to conversion into a conjugated structure. The Durham-route to polyacetylene and Dow Chemical’s Wessling-Zimmerman sulfonium precursor to poly(p-phenylenevinylene) (PPV) are two key examples, the latter being the focus of my own PhD research. The use of conjugated polymers with solubilizing side groups has led to direct solution processing, with the imagination of synthesis experts leading to a bewildering array of polymers with increasingly difficult to remember and pronounce acronyms.

The interest in conjugated polymers as synthetic metals and optical switches started to wane in the mid-1980s, largely due to, respectively, air instability and speed limitations. The invention of conjugated polymer electroluminescence in 1989 by me, Jeremy Burroughes, and Richard Friend, which used PPV as the emissive layer, shifted the field to focus on semiconductor device applications in displays, lighting, solar cells, transistors, photodiodes, sensors, lasers, and photonics. This came at the right time and rejuvenated the field. Some notable developments over the following years included the optimized synthesis of soluble polyarylenevinylenes, including the Covion Gilch route to ‘Super Yellow’; the development of a polymer display that was incorporated into ‘James Bond’s shaver of choice’, a Philishave product that was advertised in the 2002 film ‘Die Another Day’; optimization by Dow Chemicals of the Lumation® family of soluble polyfluorenes for red, green, and blue emission; development by Osram Optosemiconductors of Pictiva® displays and lighting panels using these materials; committed long-term engagement from the Sumitomo Chemical Company as well as Merck to continue this development and add further solution-processed organic semiconductor classes; and the development of inkjet printing approaches by Epson, Cambridge Display Technology (CDT), Philips, Panasonic, and Japan OLED, more recently also involving TCL in China and leading to mid-sized commercial monitors, which have been described as the ‘world’s best displays’.

The focus on a subset of polymer types was especially beneficial for performing detailed physicochemical characterization and device optimization, and I benefitted greatly from collaborations with Dow, Sumitomo, and Merck in this regard. The question of purity, and its importance, is also rather different between semiconductor classes, complicated in the molecular case by the presence of different isomers and conformers, as well as the more widely expected chemical defects and contaminants. The molecular weight, polydispersity, and end groups also need to be considered for conjugated polymers.

I would add that there should not really be any sense of competition between organic and inorganic semiconductors; they both excel in specific circumstances, and they can be combined to good effect. One could say that organic optoelectronic materials contributed to the establishment of a broader new field of soluble semiconductors, and I tend to classify my own research as ‘soluble semiconductor and molecular electronic materials and device research’, which encompasses organics but also metal oxides, hybrid perovskites, and other materials systems. There is a divide between the vacuum and solution processing communities, and some of the potential advantages of the latter remain to be fully achieved, including high-throughput roll-to-roll printing for solar, lighting, and Internet-of-Things device applications where cost is a major driver. Flexible and rollable products have been demonstrated with both organic and metal oxide semiconductors, so while some concepts were originally envisaged from an organic materials perspective, they have also been shown to be possible with inorganic semiconductors.

Similarly, areas thought to be the preserve of inorganic semiconductors have emerged in which organic semiconductors show substantial benefits. Microcavities are a good example of this where it was initially considered that the broad (vibronically structured) optical transition linewidths of organics would preclude polariton formation and related properties. In fact, organics have been shown to generate especially large Rabi splitting energies because of their high oscillator strengths and to be operable at room temperature due to their large exciton binding energies. This provides new avenues to explore in the physics and application of quantum electrodynamics.

A great interest for me in terms of semiconductors has been to exploit molecular properties to do things better or to do new things. One example is linearly polarized electroluminescence from oriented liquid crystalline polyfluorene films. Another is the achievement of room-temperature ultrastrong coupling and low-threshold lasing in microcavities. The third is conformation patterning for the definition of dielectric metamaterials.

In the future, photonics, including diverse microcavity structures and various kinds of electrically pumped lasers, are expected to be important components. Lighting, solar energy generation, and Internet of Things devices will likely need roll-to-roll printing to enable a sufficiently low cost for large-scale applications. Polymer optical fibres may also be developed beyond current automotive and local area network applications. I also sincerely hope that I will be surprised by out-of-left-field developments that are unexpected and dramatically change our thinking!

**Q2.** **One of your major achievements was inventing the first conjugated polymer light-emitting diode in 1989. How did this discovery come about and what did it tell you about the potential of the materials you were working with? Why was this an important step for the organic optoelectronics field and how did people react to the paper in** ***Nature*** **that announced it?**

When I started my PhD research, the main thrusts for research on conjugated polymers were in highly conductive synthetic metals and nonlinear optics, with the key material systems being polyacetylene, polydiacetylene, and polythiophene. None of these materials were significantly light emitting, and their suitability as conductive replacements for metals and/or nonlinear optical switches for telecoms had suffered critical setbacks that subsequently led to a cessation of most work on those topics, although polyethylenedioxythiophene (PEDOT) subsequently proved to be a very useful supplement to, and potential replacement for, the inorganic indium tin oxide films typically used as anodes.

The discovery of conjugated polymer electroluminescence that led to our fundamental patent was the result of an interest in conjugated polymer transistors. My colleague and friend Jeremy Burroughes was looking for a better dielectric to use as the gate insulator in his devices, and I suggested the possibility of using precursor-route poly(p-phenylenevinylene) (PPV), the focus of my PhD research. I knew from my earlier work that PPV could be prepared as robust pinhole-free thin films that had very low conductivity and low levels of defects and that it consequently might be a suitable material for his application. Jeremy agreed, and this led to an experiment in which he observed light emission from a simple diode structure. The first devices turned on at a bias of 3 MV/cm and showed 0.01% efficiency. We were, however, able to show that the emission was categorically electroluminescence, not a glow discharge and that it could respond at video data rates, which, together with a knowledge that the colour could be chemically tuned and an expectation that efficiencies could reach at least 1% prompted us to consider that it had potential as a technology for novel display devices based on printing. Note that this was at a time when gallium nitride LEDs were not available and the most efficient GaP LEDs operated at approximately 1% efficiency. It was also understood that conjugated polymers could emit blue light, whereas in the absence of GaN, blue light emission was problematic for inorganic LEDs, with SiC being the best option at that time. Furthermore, given the observed failure mode of the devices, in which the Al top electrode could be stripped and redeposited for emission, it was also evident that the PPV films were unexpectedly robust.

Our discovery of electroluminescence from conjugated polymers was reported in an article published in *Nature*. This paper, for which I was the corresponding author, became a ‘citation classic’, garnering more than 16,000 citations in the intervening years, and the associated US patent has been cited more than 2,000 times. Both citation numbers signal the impact that it had, with enormous excitement generated in both academia and industry. It rejuvenated the interest of researchers that had started to wane following the recognition that synthetic metals and nonlinear optical switches based on conjugated polymers were not going to lead to real-world applications. It also seems to have given a fillip to the awareness of small-molecule OLEDs, attracting a wider community to the topic than had previously engaged therewith: the citation counts for small-molecule LEDs significantly rose after the *Nature* paper became highly cited.

I spent approximately 30% of my time over the next three years meeting with interested parties from industry, government, and academic institutions to answer their questions and provide progress updates. Some of those parties were very impatient for progress, others wanted to acquire rights to the intellectual property ‘for a song’, and some were well known for what they did outside research and innovation: Hit and Run Ltd., a fund established by *Genesis* band members Phil Collins, Mike Rutherford, and Tony Banks, was one of the more memorable investors.

The fundamental patent (Friend, Burroughes, and Bradley) was challenged at the European Patent Office by Philips and Uniax but was upheld, signalling both the novelty and inventiveness of the claims and, by extension, the underlying research. This patent was subsequently one of only three shortlisted finalists from among 380,000 European Patent Office patents granted in the 1991–2000 decade that were assessed for innovation and technological impact for the European Inventor of the Year Awards 2006. The overall awardee, Peter Grünberg, subsequently won the 2007 Nobel Prize in Physics for his work on giant magneto resistance.

**Q3.** **The discovery occurred when you were only 27 years old. How did it affect your career path and what advice would you give to early career researchers who may be reading this article? Why did you subsequently choose to leave the world-famous Cavendish Laboratory in Cambridge to move to the Department of Physics at the University of Sheffield and what did you do to progress your career there?**

The discovery of conjugated polymer light-emitting diodes helped me to secure my first faculty position as an assistant lecturer in the Department of Physics (the Cavendish Laboratory). Conjugated polymer electroluminescence was a new field with great prospects, and I was ideally placed to drive the field forward. After being an assistant lecturer for three years, I reached the top of the AL grade scale, without much prospect of promotion to a lectureship any time soon. I could also see very able people in Cambridge who had not progressed as far in their academic careers as they might have if they had been willing to move, and I had been in Cambridge for ten years by that time.

My move to Sheffield in 1993 as a reader enabled me to establish my own research group from scratch and to pursue the research directions in which I was personally interested. In addition, the vice chancellor of Sheffield University, Gareth Roberts, was an inspiring leader and someone who was willing to place a great deal of trust in young faculty. I was convinced that I could build a successful group in Sheffield and, by doing so, contribute to the development of an up-and-coming physics department.

Sheffield was also an attractive part of the country, close to the Peak District and near my wife’s family in Barnsley, and a much more affordable place to live than Cambridge. Many people in Cambridge were skeptical that a move to Sheffield could be a good career step and told me that I would be throwing my career away. Within two years, I had been promoted to full professor and had a very dynamic and well-funded research group that was attracting international interest and was strongly supported by the head of department and vice chancellor.

When I got to Sheffield, I focused on establishing the credibility and visibility of my *molecular electronic materials and devices* (MEMD) group by attracting great students and postdocs, applying for research fellowships, securing funding from government agencies and industry, building research capabilities, establishing strong collaborations within Sheffield and with other institutions, writing papers, presenting at conferences and so on. Many of those who helped me to build the group as students and postdocs have gone on to impressive careers, including a pro-vice-chancellor at Cambridge University, professors at universities in the UK and elsewhere, and leaders in industry and commerce. The organic optoelectronics research activities at Sheffield have continued to thrive under the leadership of David Lidzey, with the establishment of additional research directions, a very successful start-up company, Ossila Ltd., and high visibility for the work, especially in microcavities and solar cells.

Another important factor in building the group in Sheffield was that the Dow Chemical Company selected my MEMD team as a strategic partner to work on the physicochemical characterization and device physics of polyfluorenes, which provided access to a range of state-of-the-art materials at a scale that supported extensive and detailed investigations. The papers we published on polyfluorenes, including their liquid crystalline orientation, β-phase conformation, fluorenone defects, polarized electroluminescence, charge injection and transport, and luminescence, lasing, and optical amplification, provided a fundamental platform for understanding physics and subsequent LED development over the next decade and beyond. We also worked with Philips, Sharp, Avecia, Merck, Covion, Osram, and other companies on polyarylenevinylenes and polythiophenes and built expertise in measurement techniques, including electroabsorption spectroscopy, time-of-flight photocurrent mobility measurements, temperature-dependent dark current injection and transport measurements, deep-level trap spectroscopy, and steady-state and transient photoinduced absorption and photoluminescence. The group participated in major European Union and UK collaboration projects focused principally on displays and photonics.

Over time, I recruited additional faculty members, and we organized a successful international conference, ICEL’2, in 1999 that brought people from around the world to learn more about what we were doing in Sheffield. A large body of impactful work came out of the seven years that I spent there, including important developments of polyfluorene light emitters and transport materials, understanding their materials physics and processing, demonstrating state-of-the-art performance for LEDs, advancing the optical lasing and gain properties of conjugated polymers, establishing the nonlinear optical characterization of devices, and particularly providing the first demonstrations of strongly coupled microcavities and polariton emission using organic materials, which launched a new field of research that has attracted considerable global interest. This eventually led to an approach from Imperial College to return to my *alma mater* and set up a new molecular electronic materials activity to help rejuvenate the solid state physics group at Imperial College.

It was a very happy time for me and my wife and our three children, two of whom were born in Sheffield. It was a great thrill to subsequently attend the graduation of our youngest daughter from the medical school there in 2022 and for me to receive an honorary DSc degree from the university in 2014.

In terms of advice to LSA’s early-career readers, I would say back your own ability, seize good opportunities, and do not be too easily dissuaded by others whose thoughts may be based on very different circumstances and concerns.

**Figure:**
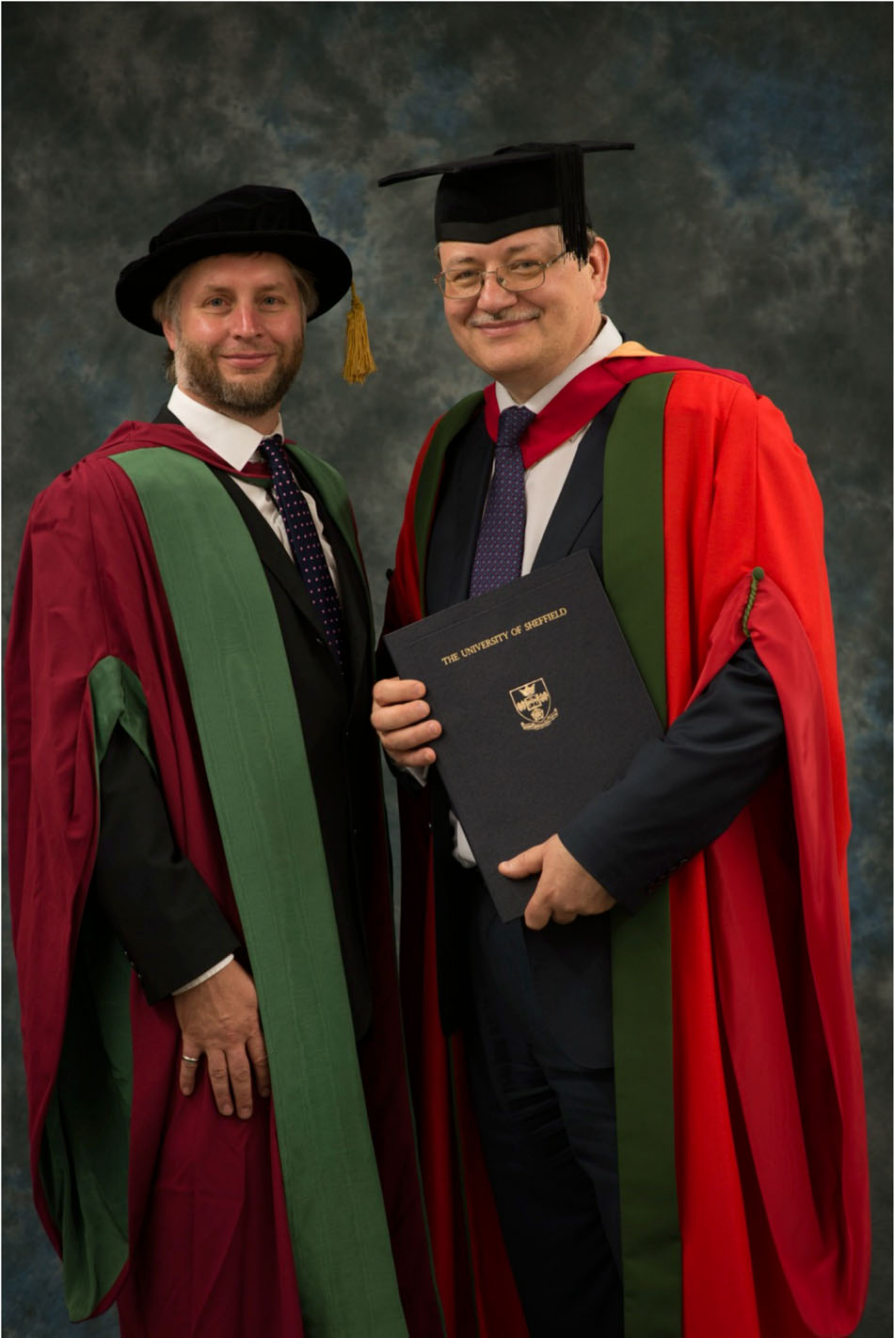
Professor Donal Bradley received an Honorary DSc from the University of Sheffield in 2014. Here he is pictured standing to the right of Professor David Lidzey before the ceremony

**Q4.** **Soon after your discovery, you co-founded Cambridge Display Technology to develop conjugated polymer displays. How did this company develop over time, and does it still exist today?**

Jeremy Burroughes and I quickly decided that we should file a patent on the invention of conjugated polymer electroluminescence, and with Richard Friend, we spoke to the University of Cambridge Technology Transfer Office. We told them that we wanted to file a patent. They asked why. We asked if they could support us in doing so, and they answered no. We, consequently, wrote the patent ourselves and paid for it to be filed. I provided much of the insight into the chemical aspects of the invention, having the most interest in and knowledge of the associated chemistry, in part because of my PhD research and my hands-on synthesis experience during my fellowship in Japan, where I made the polyarylenevinylene samples that I used for my nonlinear optics research. The first steps in developing the technology were funded through investments from the Cambridge Quantum Fund and Cambridge Research and Innovation Ltd. Several steps were taken to further exemplify the scope of the technology in terms of engineering a simple display demonstrator and working with the chemistry department team of Andrew Holmes, especially Paul Burn, on the synthesis of new materials to support chemical patterning, colour range extension, and increased efficiency and lifetime. Cambridge Display Technology was eventually founded in 1992 and involved both the physics and chemistry teams. This was a time when very little entrepreneurial activity was taking place at UK universities—a very different situation from that found today.

Cambridge Display Technology was floated on the Nasdaq index in the USA in 2004 and established a joint venture, Sumation^®^, with the Sumitomo Chemical Company in 2005 to continue the development of polyfluorenes that it had been working on with Dow before Sumitomo’s acquisition of the Dow Lumation^®^ business. The Sumitomo Chemical Company subsequently acquired CDT in 2007 for $285 M, and it became a wholly owned subsidiary. It has since operated as the *de facto* European R&D centre for the company, initially supporting the development of printed OLED displays and then diversifying into many new directions, including lighting, solar energy generation, and biosensing. Jeremy Burroughes has been the CDT CTO for 20+ years and is also a Sumitomo Chemical Fellow at the parent company in Japan.

**Q5.** **You have also co-founded and worked with several other companies interested in organic optoelectronics. What applications were they looking to develop and how have they fared? Are there any lessons that you have learned from working with companies that you would like to share with our readers?**

I did not start any companies while working in Sheffield, in part because I was too busy building the MEMD group from scratch and because of the major effort required to get CDT moving at Cambridge. I did consider starting a company with David Lidzey on strongly coupled microcavities, but we did not see a ‘killer application’. We also discussed using liquid crystalline alignment to optimize conjugated polymer lasing, but a paper was published by another group that addressed that topic, making the idea no longer novel. Plastic Logic, a Cambridge spin-off, patented liquid crystal alignment of polyfluorenes for transistors, a topic connected to work I proposed and collaborated on with researchers there. David Lidzey subsequently established the very successful laboratory equipment and materials supply company Ossila Ltd.

When I moved back to Imperial College, I teamed up with brothers John and Andrew de Mello from the chemistry department to develop medical diagnostic devices that combined organic optoelectronic light sources and photodetectors (John’s and my expertise) with microfluidics (Andrew’s expertise). We secured SBRI funding from the Biotechnology and Biological Sciences Research Council and an EPSRC grant that led to patentable results and interest from Imperial Innovations to start a company. Molecular Vision Ltd. was founded in 2001 and developed the BioLED^®^ platform with prototype devices that used organic LEDs and photodiodes. Some of the photodetectors displayed exceptional performance, and Molecular Vision was used as an impact case study for both the chemistry and physics departments in the 2014 Research Excellence Framework exercise. However, securing significant venture investment proved difficult, with the challenge of handling biological fluids in microfluidic chips combined with quantitative measurements from paired OLEDs and photodetectors being described by one venture funder as a ‘three-miracle’ problem. They explained that while they did fund ‘one-miracle’ problems and sometimes ‘two-miracle’ problems, ‘three-miracle’ problems were outside their remit!

Molecular Vision was acquired by the Abingdon Health Group, which reduced the number of ‘miracles’ required by shifting to a lateral flow geometry, working with Cambridge Display Technology, and making demonstrator devices using inorganic LEDs. The devices were Bluetooth-coupled to a mobile phone for data analysis and display, and we demonstrated an attractive point-of-care diagnostic system. After the acquisition, I served on the Abingdon Health Scientific Advisory Board, having previously served on the Molecular Vision Board as a founding director. One of the challenges that Molecular Vision faced was identifying which diagnostic tests to implement on the platform as its first product. Among others, the Cardioplex^®^ cardiac triple fluorescence marker panel was developed for emergency room triage of patients who might have suffered a heart attack.

I subsequently worked with colleagues at Imperial College to establish a limited liability partnership, C-Change LLP, that collectively supported the establishment of Solar Press (UK) Ltd., which was founded in 2009 by the UK Carbon Trust to develop roll-to-roll processed solar cell technology based on organic semiconductors combined with fullerene electron acceptors. I served on the Board of Solar Press from 2009 to 2016, and technical progress was very impressive, using blade coating to fabricate high-performance large-area solar cells.

Unfortunately, the company suffered from funding difficulties connected to a dramatic decrease in the cost of silicon solar cells, which undermined one of the major reasons for the interest in novel solar cell materials. It was (wrongly) understood that the price of silicon solar energy generation could not be significantly reduced from the plateau that it had reached a few years earlier. The introduction of a new device geometry developed by Martin Green and collaborators at the University of Queensland and a shift in manufacturing to China dramatically decreased the cost, and Solar Press and many other novel solar cell companies suffered the consequences. Solar Press struggled for a while, moving its operations to Swansea, but finally transitioned to Trameto Ltd., a company focused on integrated circuits for energy harvesting and power management, specifically addressing Internet-of-Things devices using multiple environmental energy sources.

At a similar time, CSEM Brasil was interested in working on roll-to-roll coated flexible solar cell technology, and I interacted with the team there, supporting them in applying for funding from the Brazilian National Bank for Economic and Social Development (BNDES) to establish a technology development program. This program led to the founding of Sunew, now renamed OnInn, a company that successfully commercialized flexible photovoltaics for building integration, transparent solar roofing, urban solar charger furniture, and on-vehicle transport solutions.

Other companies with which I have interacted have been strongly interested in the development of organic electroluminescent lighting, replacements for indium tin oxide transparent conductors, photodynamic therapy, flexible electronics, wearable detectors and sensors, greenhouse shading and cooling, and interactive packaging.

The lessons learned include finding ways to advance your technology before starting the company and seeking venture equity funding, identifying target applications, developing technology specifically for those targets, finding the best solutions, not just the ones you are familiar with, and understanding the complexity of the supply chains and markets you want to address.

**Q6.** **Are you associated with any current companies working in the organic optoelectronics area or do you have an interest to found other companies? If yes, please elaborate on their focus.**

I am a cofounder and director of Excyton Ltd, a UK-based company that is developing novel pixel architectures for highly efficient displays. The Excyton CEO, Dr Peter Levermore, studied for his PhD in my group at Imperial College and then worked in several companies, including UDC and Merck, and as a free-lance display technology consultant. I had been thinking for some time whether to start another company and concluded that I would only be interested in doing so if I could find a great CEO. I thought of Pete, and when I contacted him in 2018, it turned out that he was also thinking about starting a company! We discussed two ideas and settled on creating a company that would focus on enhancing the colour gamut of displays, in part responding to the requirements of the new Rec. 2020 display standard. The company was founded in 2019 and was originally called PeroLED Ltd.; it worked on developing perovskite and other solution-processed LEDs.

PeroLED received equity funding from the King Abdullah University of Science and Technology (KAUST) Innovation Fund and established a research base there. PeroLED subsequently pivoted to develop the TurboLED® technology, an innovation that offers the potential for up to 45% power savings compared to today’s leading displays through the combination of a novel pixel architecture and image processing algorithms. It was also renamed Excyton Ltd.

Excyton won an i-Zone innovation award at the 2023 Society for Information Display’s Display Week in Los Angeles for the TurboLED^®^ technology. Excyton is currently undertaking a project funded by the UK and Swiss governments to produce a demonstrator. It is also working with the Oxford Suzhou Centre for Advanced Research (OSCAR) in China. The TurboLED^®^ concept can be applied to different display technologies, including OLEDs, micro-LEDs, and Q-LEDs, significantly increasing its potential.

**Q7.** **Please highlight some of the other important progress/milestones in organic optoelectronic research since the early days on polymer light-emitting diodes? What are some of the major challenges and current research topics for organic optoelectronics? What advances have surprised or excited you? Which of these are the things you have been closely engaged with?**

This is a difficult question for someone who has worked on organic optoelectronics for forty years, as much great work has been done at universities and companies worldwide. The success of organic LEDs was followed by a strong interest in solar cells, another field for which organic semiconductors have beneficial properties. Their strong absorption coefficients across the visible and near-infrared regions support thin film formats. Direct charge generation is not as straightforward since the exciton binding energy is large, but this can be addressed by charge generation at a heterojunction interface. This subsequently led to the development of donor–acceptor blends, initially with fullerene acceptors, to provide a large heterojunction surface area, with additional structuring of the blend desirable to support charge extraction through a single phase over short distances to account for the relatively low charge carrier mobilities.

The drastic reduction in the price of silicon photovoltaics and the advent of perovskites significantly reduced interest in organic photovoltaics (OPVs), but interest has been regenerated by the switch to nonfullerene acceptors and a steady rise in AM1 power generation efficiency and cell stability. Roll-to-roll coating will likely be needed to provide a cost-benefit for OPVs, and deployment in nonconventional formats, including semitransparent cells for windows, may provide a useful niche. Roll-to-roll coating is expensive from a material consumption perspective, making it difficult to research in an academic environment. It is also imperative that supplies of materials be readily available and costs not be prohibitive for industrial development; however, without a significant market, manufacturers of materials are generally reluctant to scale up to the needed volumes and price points.

New modes of light emission in organic LEDs have also undergone exciting developments, with Chihaya Adachi and other’s thermally activated delayed fluorescence approach of particular interest for increasing OLED device efficiency. This is potentially good for sustainability, but there has been a tendency to sell increasingly larger displays rather than banking energy savings. The TurboLED^®^ pixel architecture mentioned above offers further potential for improving the sustainability of OLED (and other) displays.

Inkjet printing and/or roll-to-roll coating of LEDs may be needed for OLED lighting applications due to the required cost. This approach to fabrication has continued to develop over an extended period but has remained the preferred approach of only a small number of companies. Japan’s JOLED was the main protagonist for a while, with China’s TCL now in the vanguard. JOLED produced a series of commercial monitor displays with exceptional performance using materials from Sumitomo. The challenge is achieving high-performance levels from a smaller number of layers than would typically be used in a traditional vacuum-deposited OLED stack. To address this need, complex copolymers that combine different functionalities within a single polymer chain were developed by Sumitomo and other companies. They allow the combination of electron/hole injection, electron/hole transport, emissive state formation and radiative decay functions. Initially, these copolymers were simple alternating copolymers, such as fluorene–arylamine or fluorene–benzothiadiazole, followed by statistical copolymers with multiple functional units, eventually evolving to complex variants in which the chain architecture (specifically where the functional units are inserted into the chain) is also managed.

For roll-to-roll coating, finding alternatives to indium tin oxide (ITO) as a transparent anode layer is also desirable. ITO remains a brittle material that is not best suited for deposition and subsequent processing on a flexible substrate. This has been an interest of mine (and many others) for some time, and it would be great to see a breakthrough. The doping of organic semiconductors has also been an area that has seen significant progress, with Thomas Anthopoulos, Seth Marder, and others showing very good recent results that have allowed improved performance for solar cells and other devices.

Polariton emission within strongly coupled microcavities represents another novel mode of light emission. It was considered by those who worked on quantum-well inorganic semiconductor cavities that organic semiconductors had optical transitions that were too broad to see strong coupling, due to the vibronic levels coupled to their electronic states. Together with David Lidzey, I showed that not only does strong coupling occur but also that the polariton states formed exhibit strong emission at room temperature, opening the path to polariton-based LEDs and lasers. Ultrastrong coupling is also possible, and my team has shown that combining molecular alignment and conformation control allows exceptional coupling strengths, together with the opportunity to generate very narrow emission without the angular dispersion typical of weakly coupled microcavities. This has led to exceptional blue colour saturation for polyfluorene emitters. Oriented polyfluorene-containing cavities can also show very low lasing thresholds and have been used to make polariton LEDs. There is much more to be done in developing polariton emission for device applications, alongside the exploration of new physics.

Organic photonics more generally is an area in which I expect to see significant progress over the coming years. Electrically pumped organic lasers remain of strong interest and are covered in the next question.

**Q8.** **Do you have a view on the prospects for electrically pumped organic lasers? It has been more than 30 years since optically pumped solid-state organic lasers were first demonstrated, with many studies reported including your own work on conjugated polymers. What do you think has delayed the evolution to electrical pumping and are there any fundamental issues that cannot be solved? Do you expect that perovskite materials offer any advantages in this regard?**

The story of electrically pumped organic solid-state lasers has been complex, with many diversions and hurdles over the years. The motivation has been to replicate the versatility of semiconductor laser diodes but to use materials that can emit near-ultraviolet, visible, or near-infrared light, that can be low-threshold because of their three- or four-level vibronic optical transitions, narrow linewidth, and both tunable and short-pulse-capable. Electrically pumped optical amplifiers would also be of considerable interest, as would gain switches, wavelength converters, and so on. Organic laser gain media have historically been important in the form of dye solutions that provided a key entry point to tuneable short pulse lasers – something my father worked extensively on in the 1960s. These dyes do not, however, show strong optical gain in the solid state, with intermolecular interactions causing excitation quenching.

Solid-state organic lasers initially focused on dyes distributed within a transparent host matrix to replicate the dispersion found for solutions, but this approach proved not to be an especially attractive option, with thermal and photochemical stability issues leading to a need for frequent replacement of the gain medium. We showed that the thermal diffusion of dye molecules allowed a more uniform distribution and consequently higher loadings, but stability remained a problem. Conjugated polymers were subsequently investigated for solid-state lasing, and unlike their dye analogues, they were able to sustain strong gain in undiluted thin film formats. Amplified spontaneous emission could be easily demonstrated for spin-coated films with thicknesses greater than the cut-off for waveguiding. The addition of a scattering medium led to random lasing, whilst deposition on prefabricated dielectric and plasmon grating structures enabled the demonstration of a wide variety of 1- and 2-D distributed feedback (DFB) lasers operating in both index- and gain-coupled modes.

The relative simplicity of fabrication for these structures, especially at longer wavelengths, allowed a close connection between theory and material optimization. We showed both 1st-order in-plane and 2nd-order out-of-plane DFB laser emission for a variety of polyfluorenes and polyarylenevinylenes, fabricated compact, high-gain, grating-coupled amplifiers and demonstrated gain switching between inorganic data comm (850 nm) and visible wavelengths. The in-plane operation of structures is limited by the diffractive nature of the film edge and the inherent difficulty of defining a clean facet for these soft materials. Most work has therefore been on normal-to-plane emission. It is also not easy to laterally define waveguides, and other photonic elements—etching and implantation are generally damaging for organic gain media. We consequently developed novel molecular metamaterial approaches using spatial control over both conformation and localized orientation to address this challenge. Distributed Bragg reflector (DBR) structures (vertical microcavities with dielectric mirrors) have also been widely investigated.

The topic of optically pumped organic solid-state lasers has continued as an academic interest and can provide insight into exciton dynamics at high excitation density, which is relevant to display devices driven at high luminance, as is needed in passive matrix addressing. Such structures can also be pumped with inorganic laser diodes, and the sensitivity of the gain media to environmental factors has led to sensor development activities, including the detection of volatile amine compounds emanating from landmines. These rapidly quench the emission, and Tim Swager showed that in the case of conjugated polymers, a small number of catalytic sites can quench multiple chromophores within a single chain, resulting in high-sensitivity detection.

There are intermediate steps that could lead to useful compact light sources with electrical driving. These include LED-pumped organic solid-state lasers, something that has now been demonstrated using both inorganic and organic LEDs, notably in the group of Ifor Samuel and Graham Turnbull. This approach would be the equivalent of inorganic solid-state diode-pumped gain media such as titanium sapphire, which have proven highly useful, although the application space would be very different.

The specific grand challenge of electrically pumped organic and perovskite hybrid semiconductor lasers requires optimization of many properties to adequately manage optical and electrical device requirements. A simultaneous reduction in the exciton density needed for stimulated emission and an increase in the density achievable with electrical pumping is necessary to advance the state of the art. This requires a separation of functions or concurrent optimization of materials for multiple functions – a challenging task. Minimization of exciton quenching from exciton–exciton, exciton–charge, and exciton–triplet interactions and limitation of optical losses due to lasing mode overlap with lossy elements of the structure, together with control over thermal and photooxidative degradation processes, are needed. These issues apply equally to organics and hybrid perovskites, in the latter case with the addition of Auger recombination losses. Perovskites can achieve higher charge carrier mobilities of ~10 cm^2^/Vs vs. ≤ 0.1 cm^2^/Vs for many organic emitters, which allows higher injection currents, however only where material stability limitations do not interfere. Barry Rand and his colleagues at Princeton are driving this topic forward with impressive results. Films with higher refractive indices support in-plane propagation at lower thicknesses and can thus reduce normal-to-plane carrier transit times, potentially reducing lattice relaxation and consequent absorption losses. Graded index profiles may also be possible for polymer-based structures through spatial control of chain conformation in the vertical direction.

To further address the electrical-pumping challenge, several lessons learned from across the past decade and beyond should be considered. Key factors include the following: (i) Light-emitting materials optimized for display applications do not necessarily have a full coincidence of attributes needed for lasing. Materials that work well as steady-state light emitters can have states that reduce exciton density in the short time scales needed for stimulated emission. For example, the complex copolymers developed by Dow, CDT, and Sumitomo often show excellent steady-state luminescence and electroluminescence, but their optical gain characteristics are generally poor. Charge-separated states formed at short times quench excitons and have absorptive optical transitions in the spectral window for gain, increasing thresholds and/or preventing population inversion altogether. Exciton confinement helps to limit this effect and could be used to design organic semiconductor systems specifically for lasing. It will be interesting to see whether the synthesis of specific optical gain materials for lasing will be addressed more widely in the coming years. (ii) The PLQE values of organic thin films are typically ≤50%, and the degree to which this is a purity and/or photostability issue remains largely unknown. Microstructure plays a key role here, with different ways of processing the same chemical structure having a large impact. For example, generating low *β*-phase fractions in PFO significantly enhanced the PLQE (from ~50 to ≥70%). Systematic studies on how to increase and stabilize the PLQE should be undertaken for selected target materials. (iii) Optimizing charge injection and transport without quenching excitons has long been an issue. Achieving high excitation densities requires significant charge injection, with space-charge-limited current densities under perfect ohmic injection conditions determined by the charge carrier mobility. This led us to an early interest in optimizing charge carrier mobility alongside luminescence efficiency, as demonstrated for blue-emission polyfluorenes, yielding some of the lowest optically pumped laser thresholds and best slope efficiencies reported to date, even after more than 15 years. We identified the benefits of improving intrachain transport via chain-extended conformations and creating sparse local sites that support efficient interchain transfer, approaches that others have followed. New emission materials should be developed to further advance this strategy. (iv) It is important to actively manage triplet states and avoid trapped charges. Their long-lived intragap absorption losses generally prevent CW operation and increase thresholds; additionally, triplet states generate singlet oxygen, promoting degradation. My research team has used carrier-density-dependent mobility measurements and electroabsorption/charge modulation spectroscopy to study charge traps, and more work should be done on the best ways to limit their presence. (v) The optical structure should be engineered to reduce overlap between lasing modes and lossy electrodes. In the case of sufficient carrier lifetimes and/or carrier/exciton diffusion lengths, there is the option to spatially separate injection and feedback. This is done to some degree in the exciting structures reported by Chihaya Adachi and might be even more applicable for perovskites.

Another challenge for those interested in working on electrically pumped organic lasers has been fallout from the discredited claims of Bell Lab’s researcher Jan-Hendrik Schön and his coworkers. This situation led to an aversion by many to becoming involved in a topic that was considered tainted. This has also led to difficulty in publishing papers, with the criteria to claim organic lasing established in a way that would preclude many inorganic lasers that are currently widely accepted as such.

There are interesting reports in the literature of both microcavity DBR (Xingyuan Liu, CIOMP) and DFB (Chihaya Adachi, Kyu-dai) electrically pumped organic laser structures, but these have struggled to be accepted at face value. The reported DBR structures, if not actual lasers, would nevertheless be of considerable interest as high-quality resonant cavity LEDs, and the reported DFB structures demonstrated a tour-de-force engineering achievement to combine all the expected elements in a single structure. In both cases, further development and optimization are likely to be possible.

Some researchers have argued that electrical pumping is not possible for organic semiconductors. It is true that there are important matters still to be addressed, but for that very reason, the challenge has stimulated interest to tackle some difficult questions of our knowledge and understanding. It should, also, be recalled that strong voices once argued that organic LEDs would never be sufficiently robust for applications, and we now have excellent OLED displays all around us. Similarly, other experts stated that strong coupling would not be possible for organic microcavities, but it very clearly is. Therefore, when we are told that something cannot be done, we should not be afraid to challenge that view, as sometimes the assumptions behind it prove to be wrong.

If electrically pumped organic and/or other solution-processed semiconductor laser diodes can be successfully developed, there are interesting opportunities for their use in chip-to-chip optical communications, in combination with polymer optical fibres for data comms, environmental and health-related sensing and imaging, spectroscopy, and so on.

In addition to the laser diode structures discussed above, there is also interest in light-emitting transistors and strongly coupled microcavity lasing. In the latter case, cavities that operate simultaneously in strong and ultrastrong coupling via β-phase and glassy exciton states within liquid crystalline-oriented films have shown new low threshold levels – a nice example of molecular conformation and alignment being used to subtly control optical characteristics. Electrically pumped resonant cavity LEDs of this type have been demonstrated by Malte Gather and his group.

**Q9.** **You have worked in several places outside the UK during your career and have developed extensive research collaborations across the globe. You were a postdoctoral research fellow in Japan after you completed your PhD, have held adjunct positions in China, have collaborated extensively with institutions and companies in Japan, USA, Europe, Korea, China, and Brazil, and you now work in Saudi Arabia. What motivated you to work in and with these countries and regions and what do you see as the benefits of such research experiences?**

I believe that science is a social good that can help to bring the world together, and I have very much enjoyed the privilege of working with researchers worldwide and visiting many countries in the process. My experience is that I have learned more from others than they have learned from me, and I have been fascinated to hear about the rich history and culture of the places I have visited and have enjoyed their culinary specialties. I have also experienced generous hospitality and friendship from so many people.

My interests in research have had a strong application focus, and I have enjoyed working with companies developing materials for applications, including displays, communications, electronics, energy generation, and health diagnostics. In particular, I have built strong, mutually beneficial partnerships with several chemical companies that have provided a significant boost to my research programs. These include Sumitomo, Dow, Merck, and DIC Corporation. I have also collaborated extensively with academic materials synthesis teams at Cambridge, Imperial College, Oxford, Changchun Institute of Applied Chemistry, Kyushu University, KAUST, NanjingTech, MPI Stuttgart, University of Strathclyde, University of Leipzig, University of Mons, University of Potsdam, and others.

My first visit to Japan was when I was a PhD student and attended the International Conference on Synthetic Metals (ICSM) in Kyoto in 1986. This was a fantastic experience, and I subsequently decided to apply for a Toshiba Fellowship at the end of my PhD to work at the Toshiba R&D Center in Kawasaki. I also made great contacts with Tokyo University (To-dai) and Kyushu University (Kyu-dai), both prominent institutions for organic semiconductor research. The connections I established led to long-term collaborations and funding for my research from the Japanese NEDO program, Toshiba Corporation, Sumitomo Chemicals, and others. The commercial development of organic LEDs for displays is inextricably linked to the activities of Professors Saito and Tsutsui and their students and postdocs at Kyushu University. Over time, display technology has shifted its locus to Korea (LG and Samsung) and China (BOE, TCL, etc.), but Japanese companies such as Sumitomo have continued to be very influential, especially in relation to solution-processed materials development. Solar cell development has also been a strong focus for Korea, China, and Brazil.

**Q10. You have supervised PhD students and mentored postdoctoral researchers who joined your group from numerous countries of the world, including China, Korea, Japan, India, USA, and many countries in Europe. How did you select those students and postdocs to join your group and how did you seek to support them to establish career paths in research and innovation?**

I have been fortunate to supervise and mentor approximately 100 students and postdocs from across the globe. Many of them essentially selected me by writing to me, approaching me at talks and conferences or during visits, and applying for advertised positions in my group. Their distribution then reflects the distribution of the venues I attended as well as the visibility of research similar to my own in the locations where they have studied or undertaken previous research. In interviews, I like to understand their depth of knowledge and independence of thought and to explore their ability to transfer knowledge and experience to different scenarios. I was also looking for people whom I thought would have a positive influence on the group dynamic in terms of expertise and personality. Sometimes, one student or postdoc leads to a pipeline of students and postdocs from a particular location through peer-to-peer recommendations.

When students, postdocs, and visitors have joined my group, I have supported them through providing interesting problems to work on, with interesting people to work with. I have also sought to provide opportunities to gain experience working away from my group as an intern or collaborator with another laboratory. I have further encouraged them to attend and present at conferences and to write papers. After they left the group, I kept in touch with a wide range of them and provided support and advice when requested. It has also been great to learn about the many directions they have subsequently pursued. I frequently write references and I have also recommended people to headhunters or to colleagues looking to fill positions. In all of this, I have encouraged my group members to continue to learn new things wherever they go.

**Figure:**
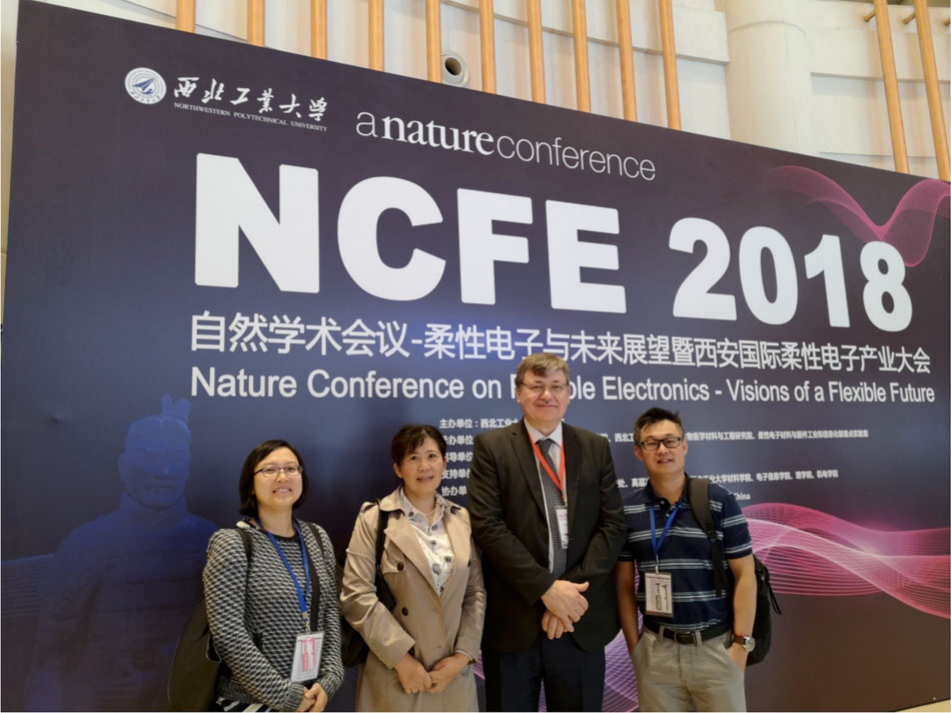
Prof. Donal Bradley met with a former PhD student and two former postdocs at the Nature Conference on Flexible Electronics in Xi’an in October 2018, namely Dr. Boon Kar Yap, Dr. Ruidong Xia (to the left, both at South China University of Technology) and Dr. Jingsong Huang (to the right, OSCAR)

**Q11.** **You are not only a successful scientist but have also been a long-term academic administrator, serving as a Head of Department, Centre Director, and Vice-Rector for Research at Imperial College, Head of the Mathematical, Physical, and Life Sciences Division at the University of Oxford, Vice-President for Research at King Abdullah University of Science and Technology and now Vice-President for Research and Innovation at NEOM U and Executive Director of the NEOM ERI Foundation. What motivated your involvement in these major administrative roles and how has it affected your own research activities? What has been your approach to supporting the establishment of strong research and innovation activities in the institutions where you have worked, which include three of the top ten ranked universities in the world?**

My research interests are quite diverse, encompassing applied physics, chemistry, materials science, device engineering, and elements of the life sciences. Molecular science provides a connecting thread that spans traditional disciplines and departments. Molecular science needs many different disciplinary inputs to progress, and I have generally been interested, as a consequence, in collaboration and the sharing of resources and facilities to bring together the different elements that create a critical mass of expertise and facilities to tackle ‘bigger picture’ questions. This requires a certain culture and a leadership perspective that is not strongly discipline-focused. Disciplinary strength is still needed and needs to be nurtured, but it is also necessary to bring people together to work outside their disciplinary constraints.

As I have progressed in my career, I have tried to understand how to develop that approach and have taken on roles of responsibility to be a part of conversations that shape such opportunities within an institution. When I was recruited back to the Imperial College Physics Department by John Pendry to lead a new initiative in organic semiconductor device physics in 2000, one of the reasons to accept the offer was that I was convinced that Imperial would be a place where I could establish an activity with the requisite scope and scale. I founded the Centre for Plastic Electronics to establish a shared research facility and brought together a group of physicists, chemists, and materials scientists to work on the science and engineering of organic optoelectronic devices. The CPE was a great success and helped propel the careers of many researchers, gaining global visibility and recognition for its research and training activities. So far, four of the CPE faculty members have been elected as Fellows of the Royal Society, the UK’s National Academy of Science, Engineering, and Medicine, and many students, postdocs, and faculty members had their work recognized by awards and prizes and developed outstanding career paths following their time in the CPE. The CPE has also established many fruitful collaborations with industry and academic institutions worldwide. Our efforts to build the CPE significantly benefited from the support of senior colleagues, particularly Peter Knight, who understood our motivations and provided strong support in progressing these activities.

**Figure:**
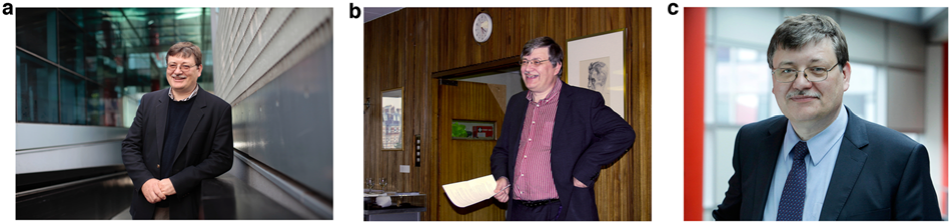
**a** Director of the Centre for Plastic Electronics, Imperial College (2009–15). **b** Head of the Department of Physics, Imperial College (2005-08). **c** Vice-Rector for Research at Imperial College (2011-15)

I have been involved in major administrative roles since 2005 when I became head of the large physics department at Imperial College, and I have held such roles at four different academic institutions, including NEOM U, where I am currently Vice-President for Research and Innovation. Each role has brought opportunities to impact the way that things get done and to help progress initiatives beneficial to the institution and its faculty. Some of the highlights beyond the CPE include OSCAR discussed below and the Circular Carbon and Future of Semiconductors Initiatives at the King Abdullah University of Science and Technology (KAUST).

The impact on my own research activities has been substantial; these are not roles that can be undertaken without serious commitment and focus. I have, however, continued to pursue a serious personal interest in research and publish papers. One way that I have been able to do that is to work collaboratively with colleagues and a great bunch of students and postdocs who embraced approaches to research in which doing things together allows outcomes that are greater than ‘the sum of the parts.’ My interest in topics at the intersection of applied physics, materials science and chemistry, and device engineering, combined with a commitment to shared infrastructure, critical mass in ‘ideas to outcomes’ delivery, and training at scale, has also helped. In addition, I have spent much time in the evening, on weekends, and during holidays working on proposals and writing and editing papers, posters, and presentations.

**Q12**. **As Head of the Mathematical, Physical, and Life Sciences Division at the University of Oxford, you supported the setting up of the Oxford Suzhou Centre for Advanced Research (OSCAR) in 2018. This was the first overseas research institute in the 900-year history of Oxford University. What was the main interest for Oxford University to establish OSCAR and how has it progressed over its first five years?**

November 2023 saw the fifth birthday celebrations of OSCAR in Suzhou, which I was delighted to attend in my role as a visiting academician there. Shortly before I joined Oxford as Head of the Mathematical, Physical, and Life Sciences Division in 2015, a team from the Suzhou Industrial Park visited Oxford seeking to progress a proposal that had been first mooted several years earlier to establish an Oxford University presence in Suzhou. At that time, I already had extensive experience working with institutions in China, specifically Jiangsu Province, and was asked by the university to take responsibility for steering the proposal through the internal approval process to determine whether to proceed. With my colleague and good friend, Chemical Engineering Professor Zhanfeng Cui, the current director of OSCAR, we asked the question of the wider university and its leadership.

I was not then aware that this would be the first overseas research institute for Oxford, but I thought that the case for Oxford to progress the establishment of OSCAR was relatively straightforward. It was clear that the scientific progress, innovation efforts, and economic development of China were making great strides and placing China in an increasingly important position within the global research and innovation ecosystem. This was very true for my own research interests in display technologies and solar energy generation and in many other topics. I considered having greater knowledge of China, its ambitions, and the successes of its research programs beneficial for the UK and Oxford University. I also considered that appropriate participation in this progress would help to build good relationships and would offer many opportunities for Oxford researchers to pursue their more application-focused research interests at scale. The strap line for OSCAR was *The Future of Science is Global*, which strongly encapsulated the motivation to proceed. It was also important that Oxford researchers spend time in OSCAR, that they should work on projects that did not duplicate efforts in Oxford, and that they should engage with local institutions, agencies, and companies to make the activity at OSCAR distinct and properly embedded in the Suzhou Industrial Park.

Professor Cui has done an excellent job in steering OSCAR through the COVID-19 pandemic and increasing engagement with companies in China, and his own research efforts with colleagues have led to the development of a commercial rapid diagnostic test for the COVID-19 virus that was rolled out in airports and used by the UK Premier League to ensure the safety of football teams.

OSCAR has been a great success and offers an important example of global organizations working together to mutual benefit. Since its inception, additional Oxford faculty members have become involved, and more partnerships have been established within China, including innovation technology centres, which are cofounded by external organizations in collaboration with OSCAR. I am pleased to have played a pivotal role in the establishment of OSCAR and still enjoy working with colleagues at the OSCAR Optoelectronic Technologies Laboratory under the guidance of Paul Stavrinou and Jingsong Huang, with a focus on LEDs, microcavities, and laser devices.

**Figure:**
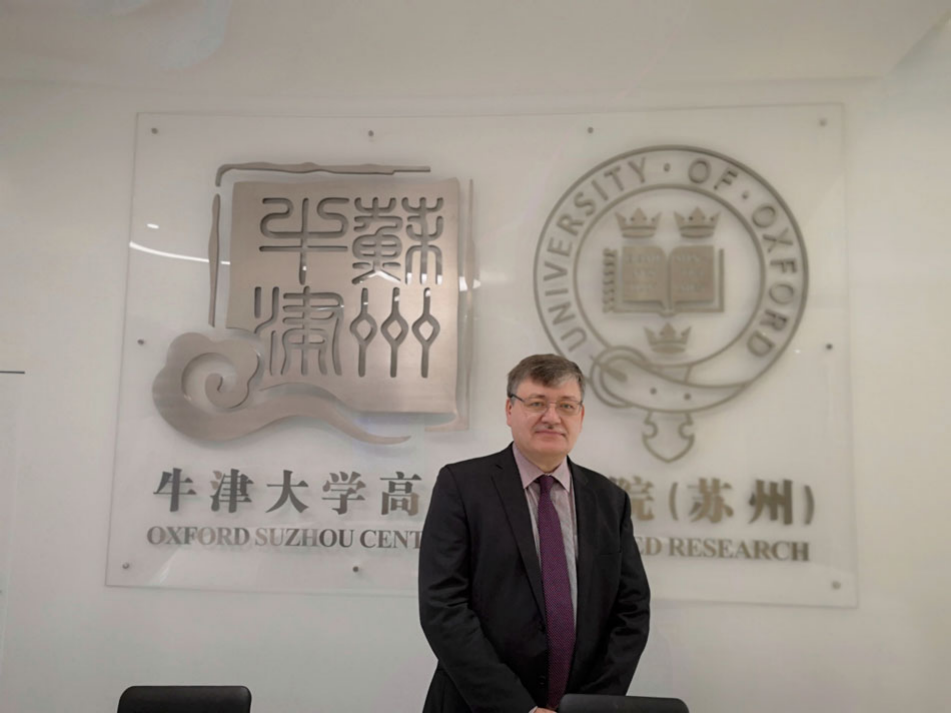
Prof. Donal Bradley at the opening ceremony of Oxford Suzhou Centre for Advanced Research in November 2018

**Q13. You currently work as Executive Director of the NEOM Education, Research, and Innovation Foundation and as the NEOM U Vice-President for Research and Innovation. Please tell us about this project and any research plans that relate to optoelectronics?**

NEOM was established to promote sustainable development and diversification of the Saudi economy. Its name comes from the Greek word *neos*, for new, and the first letter of the Arabic word *mustaqbal*, meaning future. NEOM is the size of Belgium, has a 450 km coastline along the Red Sea and Gulf of Aqaba, is within 6 hours of air travel for 40% of the world’s population, and is on the major shipping route that accounts for 15% of the world’s cargo through the Suez Canal. The regions already under development at NEOM include the LINE, a linear city designed to eventually house nine million people; Oxagon, the port city at the heart of NEOM’s industrial zone; Trojena, a mountain resort selected to host the 2029 Asian Winter Games; and Sindalah, a luxury resort island that will attract yachts and other visitors from across the globe.

In relation to optoelectronics, NEOM will deploy many devices and systems that utilize optoelectronics, including but not limited to solar energy generation, the Internet of Things, sensing and imaging, displays, solid-state lighting, communications, virtual and augmented reality, entertainment, autonomous vehicles, and digital health. NEOM U will have programs addressing many of these topics, including research and innovation activities. The established ERI Foundation Applied Research Institutes in Ocean Science and Solutions (OSSARI), Hydrogen and e-fuels (HEFARI), and Future of Urban Livability (FULARI) will also engage with optoelectronics for data collection and display, digital twin development, etc. NEOM sectors and industries will similarly rely on and develop optimized optoelectronic platforms for their activities. Consequently, optoelectronics will thrive as a technology requirement for NEOM, and new devices and systems will be developed and deployed over time.

**Q14. Several members of your family studied Physics at university. Do you think there is a particular reason that was the case?**

My father, Dan Bradley, wife, Bev, and son, Conor, are all physics graduates like me. I met my wife at Imperial College when we were both undergraduates in the Blackett Laboratory, so serendipity explains that coincidence.

My father pursued his undergraduate degree at Birkbeck College in London while simultaneously teaching in secondary schools and went on to become head of the department at Queens University Belfast and then Imperial in the 1960s and 1970s. He had a major influence on laser physics in the UK, establishing Queens University Belfast as a formidable centre for laser research in the late 1960s and then transferring a significant fraction of that activity to Imperial College in 1973. His students and postdocs went on to become leaders in many laser groups worldwide, including in China. He is probably best known for his work on short-pulse laser development and associated measurement systems, including the streak camera. As a child, I met several Nobel Prize-winning laser physicists, including Aleksandr Prokhorov, Nikolay Basov, and Art Schalow. When I later chose to study physics at university, Imperial seemed the obvious choice, in part I am sure because I was very familiar with the location from when my father worked there.

Like my wife and I, Conor is a graduate of the Imperial College physics department. While all three of our children were good at physics at school, Conor was the one who chose to study physics at university, and he liked the idea of following in his parents’ footsteps at the Blackett Laboratory, perhaps for reasons similar to my own.

**Figure:**
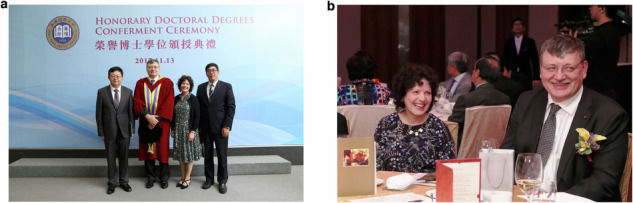
Prof. Donal Bradley and his wife Bev at Hong Kong Baptist University (HKBU) Honorary DSc Ceremony in November 2017. **a** With (left) Professor Daping Chu (University of Cambridge), Bev, and (right) Professor Zhanfeng Cui (University of Oxford). **b** HKBU Honorary DSc Council Dinner

**Q15. Among your friends and colleagues, it is known that you have been enjoying sea fishing during your time in KSA. We hear there is an interesting story that you identified a new species of Sea Bream in the Red Sea. Please let us know how this came about and what you enjoy most about fishing in KSA.**

I find fishing a great way to relax. It has been wonderful to be so close to the Red Sea during my time in Saudi Arabia, both at KAUST and now in NEOM. The Red Sea and Gulf of Aqaba contain a large variety of marine life, and I have caught many different species of fish, mainly from the shore but also from boats. My largest specimens were a 14 kg barracuda from the shore and a18 kg giant trevally and 25 kg swordfish from a boat. In all, I have caught close to thirty different varieties of fish in the Red Sea and Gulf of Aqaba.

The Red Sea coastline is a very beautiful location, with abundant coral reefs, coral flats, and beaches and a myriad of islands, and its sunny weather makes it an excellent place to spend time fishing, although it can be windy at times. I developed an interest in the sea and its fauna at a young age, with my mother, who trained as a botanist, spending time with me and my siblings exploring rock pools along the shore when we went on holiday. I started fishing at the age of four or five from the Roundstone pier in Connemara on the Atlantic west coast of Ireland and spent many summers there fishing in the lakes, rivers and along the seashore. I had not subsequently been fishing to any great degree for numerous years before my move to Saudi Arabia, but it provided a fantastic opportunity to return to something I had greatly enjoyed in my youth.

The Latin name of the new species of sea bream is *Acanthopagrus oconnorae*, derived from my mother’s maiden name, O’Connor. I named it for my mother’s 90th birthday, and its common name is Bev Bradley’s bream, named after my wife. It is unusual to find a new species of fish, especially one that is this large, approximately 30 cm in length, in a part of the world that has been extensively studied by marine biologists for more than 200 years.

**Figure:**
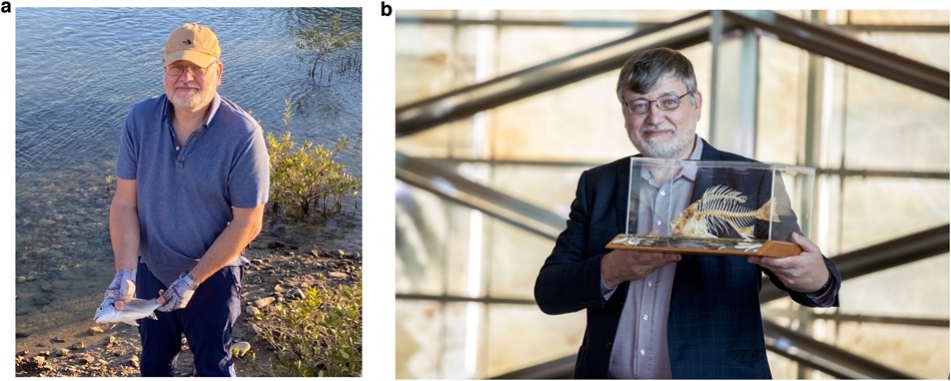
**a** On the shore of the Red Sea with a freshly caught *Acanthopagrus oconnorae* specimen. **b** Presentation of a mounted *Acanthopagrus oconnorae* skeleton to KAUST

I first caught *Acanthopagrus oconnorae* in relatively shallow water close to mangrove thickets. This is possibly not a place where commercial fishing occurs and would not have been a focus for many marine biologists attracted by the biodiversity of the Red Sea’s famous coral reefs. This might be one reason that it was not identified before. It is also very similar in appearance to the well-known *Acanthopagrus berda*, the goldsilk, or picnic seabream. I had been regularly catching seabream, but after a while started to notice that there were two different types in my catch, the goldsilk and another, that did not truly match anything reported in my books or online. It had a different head shape, a noticeable dark spot on the gill cover, and other colour variations around the fins. I asked my colleagues at the KAUST Red Sea Research Center whether this could be a new species, and following a genetic analysis, they confirmed that it was a new species. Good observational skills and a questioning mind can be very productive tools in many circumstances! I like to refer to my fishing endeavors as *Piscatorial Ichthyology*, the study of fish by fishing. By the way, I have not been fishing in China, so that would certainly be an ambition to fulfil in the future.

**Q16. Finally, have you heard of the journal Light: Science & Applications? There is a Light Office in Thuwal, within the King Abdullah University of Science and Technology (KAUST) where you worked as Vice President for Research before joining NEOM. A** **Special Issue****on Optoelectronics at KAUST has just been launched and is seeking submissions from current faculty and alumni. Would you be willing to contribute an article to this Special Issue?**

I know *Light: Science & Applications* as a very successful journal within the Springer Nature portfolio, supported by the Changchun Institute of Optics, Fine Mechanics, and Physics, an institute that I have visited and where I am pleased to collaborate. I am a founding and chief advisory editor of *npj Flexible Electronics*, a journal co-founded with Academician Wei Huang, in part guided and inspired by the success of *Light: Science & Applications*. I had not heard previously about the LSA Office in KAUST, but I am pleased to hear of its establishment under Yating Wan and to learn of the special issue being coedited by Yating together with my friends and colleagues Boon Ooi, Qiaoqiang Gan, and Xiaohang Li. I would be happy to submit an article, time permitting, and am already considering what might be a suitable topic on which to write a paper to send. I have previously published with Boon Ooi on the use of biological samples from the mantle of the Red Sea giant clam, *Tridacna gigas*, for large bandwidth wavelength conversion in ultrafast communications.

**Light Special correspondent**

*Dr Y. Hu received a BSc from Beijing Normal University in physics and a PhD from Beijing Institute of Technology. She was a professor of Physics at Capital Normal University and a visiting scholar at RPI USA. Her research interest is mainly in the THz field*.

